# Splenic arterial neurovascular bundle stimulation in esophagectomy: A feasibility and safety prospective cohort study

**DOI:** 10.3389/fnins.2022.1088628

**Published:** 2022-12-22

**Authors:** David J. Brinkman, Isha Gupta, Paul B. Matteucci, Sebastien Ouchouche, Wouter J. de Jonge, Robert W. Coatney, Tariqus Salam, Daniel J. Chew, Eric Irwin, R. Firat Yazicioglu, Grard A. P. Nieuwenhuizen, Margriet J. Vervoordeldonk, Misha D. P. Luyer

**Affiliations:** ^1^Department of Surgery, Catharina Hospital, Eindhoven, Netherlands; ^2^Tytgat Institute for Liver and Intestinal Research, Amsterdam UMC, University of Amsterdam, Amsterdam, Netherlands; ^3^Galvani Bioelectronics, Stevenage, United Kingdom

**Keywords:** esophagectomy, stimulation, splenic artery, feasibility, Doppler

## Abstract

**Introduction:**

The autonomic nervous system is a key regulator of inflammation. Electrical stimulation of the vagus nerve has been shown to have some preclinical efficacy. However, only a few clinical studies have been reported to treat inflammatory diseases. The present study evaluates, for the first time, neuromodulation of the splenic arterial neurovascular bundle (SpA NVB) in patients undergoing minimally invasive esophagectomy (MIE), in which the SpA NVB is exposed as part of the procedure.

**Methods:**

This single-center, single-arm study enrolled 13 patients undergoing MIE. During the abdominal phase of the MIE, a novel cuff was placed around the SpA NVB, and stimulation was applied. The primary endpoint was the feasibility and safety of cuff application and removal. A secondary endpoint included the impact of stimulation on SpA blood flow changes during the stimulation, and an exploratory point was C-reactive protein (CRP) levels on postoperative day (POD) 2 and 3.

**Results:**

All patients successfully underwent placement, stimulation, and removal of the cuff on the SpA NVB with no adverse events related to the investigational procedure. Stimulation was associated with an overall reduction in splenic arterial blood flow but not with changes in blood pressure or heart rate. When compared to historic Propensity Score Matched (PSM) controls, CRP levels on POD2 (124 vs. 197 mg/ml, *p* = 0.032) and POD3 (151 vs. 221 mg/ml, *p* = 0.033) were lower in patients receiving stimulation.

**Conclusion:**

This first-in-human study demonstrated for the first time that applying a cuff around the SpA NVB and subsequent stimulation is safe, feasible, and may have an effect on the postoperative inflammatory response following MIE. These findings suggest that SpA NVB stimulation may offer a new method for immunomodulatory therapy in acute or chronic inflammatory conditions.

## Introduction

The autonomic nervous system has a critical role in regulating immune function, *via* the “inflammatory reflex” (Tracey, 2002; Koopman et al., 2017). The efferent arm of the inflammatory reflex controls the systemic immune responses *via* nerves, traveling with the splenic artery (SpA) (Huston et al., 2006; Rosas-Ballina et al., 2008). These nerves form a neurovascular bundle (NVB) (Swirski et al., 2009) which contains fibers that enter the splenic parenchyma where neurotransmitters, in particular catecholamines, are released from nerve endings that subsequently modulate immune cells (Swirski et al., 2009). Exogenous electrical activation of neural pathways targeting the spleen has been shown to modulate systemic cytokine production in rodents, using either upstream activation of the cervical vagus nerve, or near end-organ activation of the SpA NVB (Borovikova et al., 2000; Rosas-Ballina et al., 2008; Vida et al., 2011; Guyot et al., 2019; Kressel et al., 2020). This mechanism has also been investigated in large animal studies where acute activation of the SpA NVB, in terminally anesthetized pigs, has been shown to reduce the production of tumor necrosis factor α and interleukin 6 following endotoxemia (Donega et al., 2021). In this model, SpN stimulation (SpNS) has also been shown to produce amplitude- and frequency-dependent changes in SpA blood flow, which are directly correlated to nerve activation.

Clinical evidence from feasibility studies of electrical cervical vagus nerve stimulation suggests that such interventions have beneficial effects on inflammatory and clinical parameters of patients with rheumatoid arthritis and Crohn’s disease (Bonaz et al., 2016; Koopman et al., 2016; Genovese et al., 2020). While these clinical findings are supportive of the role of the inflammatory cascade in these diseases, application of this therapy for inflammatory diseases is to date not approved as standard of care. Challenges with VNS at the cervical level range from off-target effects such as bradycardia and hoarseness or more serious side-effects including effects on respiration, heart rate (HR) variability or stimulation-induced obstructive sleep apnea (Nagarajan et al., 2003; Zaaimi et al., 2005; Pruvost et al., 2006; Khurana et al., 2007). Such off-target affects are due to the activation of low threshold cervical vagal fibers to the larynx, pharynx, heart, and lungs (Nicolai et al., 2020). Activation of the sympathetic path directly at SpN may avoid such side effects and allow the full neuro-immunomodulatory effect to be accessed.

To further evaluate the potential of SpNS in humans, a cuff was developed that can accommodate the pulsating features of the SpA and can effectively stimulate the SpA NVB in humans. The laparoscopic placement of the cuff and assessment of stimulation parameters were first evaluated in farm pigs (Sokal et al., 2021).

In the current study, we evaluated the feasibility and safety of temporarily placing the cuff around the SpA NVB and delivering acute stimulation in patients undergoing minimally invasive esophagectomy (MIE). This procedure was selected for this evaluation since it requires exposure of the splenic NVB as part of the procedure and cuff placement can occur with little or no additional dissection of the SpA. In addition, MIE is a surgery with a known postoperative inflammatory response. Studies suggest that the postsurgical inflammatory response is associated with increased postoperative morbidity and mortality as reviewed by Straatman et al. (2018) and attenuation of an excessive inflammatory response through SpNS may therefore reduce morbidity and mortality following MIE.

## Materials and methods

### Study design and patients

This intra-operative study was a first-in-human, single-arm open-label, single-center trial that was performed at the Catharina Hospital Eindhoven, a district teaching hospital in the Netherlands. The principles of Good Clinical Practice and Declaration of Helsinki were followed. The study protocol was approved by the Medical Ethics Committees United (MEC-U, Nieuwegein, Netherlands) under reference numbers NL70757.100.73 and R19.048. The institutional review board of the Catharina Hospital also approved the protocol and the informed consent. All patients provided written informed consent before enrollment in the study. The study protocol was registered with Clinicaltrials.gov under NCT04171011.

At the point in the standard MIE when the lymphadenectomy was performed, the splenic NVB was being isolated, and an investigational cuff was implanted laparoscopically around the NVB and connected to an external pulse generator. An ultrasound transducer was introduced to the abdomen and placed on the NVB to visualize splenic arterial BF during stimulation. The NVB was stimulated two to four times (with intervening pauses to observe post-stimulation recovery) using parameters selected to cause a change in splenic arterial blood flow (a marker of NVB activation). Stimulation parameters were adjusted over the course of the study based on experience with prior study participants. Blood samples were taken before, and at certain pre-defined time points after, stimulation and recovery. The cuff and ultrasound transducer were removed after completion of stimulation, and the surgery was continued. Surgeon experience applying and removing the cuff was documented, in addition to the responses to NVB stimulation. Postoperative safety was followed through 7-days post-surgery (or day of discharge if earlier).

Safety was reviewed by the principal investigator (ML) and a sponsor-appointed Medical Monitor (EI) after three and six patients had completed the study. Additionally, all patients were monitored for adverse events (AE) and were followed until the AE events were resolved.

Participants were eligible for inclusion if they underwent MIE. Other inclusion criteria were age equal or above 21, male or female of non-reproductive potential, a confirmed SpA loop on preoperative computed tomography angiography (CTA), and normal blood pressure or hypertension managed with medication and who were otherwise deemed fit for surgery. Exclusion criteria were previous splenectomy, an existing implantable device, active pancreatitis, or history of severe pancreatitis with complications, hepatic or splenic disease, use of oral steroids 4 weeks prior to inclusion, current use immunosuppressive agents or biologicals, and use of anticoagulants within 1 week of surgery. Potential study participants were pre-screened by the treating physician or a member of the research team as they were referred for curative treatment of esophageal carcinoma. When all eligibility criteria were met, the patient was informed and written informed consent was obtained by the treating physician (GN or ML). During a run-in period at the start of the study, three patients were included to optimize the techniques for cuff placement and ultrasound recording. Outcome analysis of these run-in patients was similar as for the other patients.

### Neuromodulation system

**FIGURE 1:**
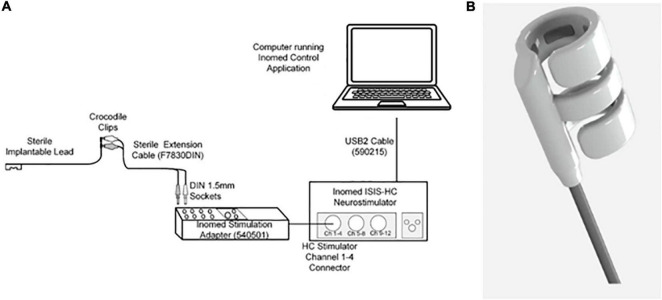
**(A)** Stimulation set-up. A laptop with the stimulation software (Inomed Control Application) was connected to the Inomed Neurostimulator (Model 504185). *Via* the extension cable, the neurostimulator was connected to the sterile implantable cuff. **(B)** The distal end of the cuff. This is a cuff which was composed of two arms with electrodes and one middle arm, designed to fit around the SpA NVB.

The Galvani stimulation system (Galvani System) consists of an implantable cuff connected to a 95 cm lead (models 10303-95 and 10305-95, investigational product; supplied sterile), an extension cable (model F7830DIN, Fiab SpA, Italy supplied sterile), and an external pulse generator [Inomed ISIS-HC Neurostimulator (Model 504185); Inomed Medizintechnik GmbH, Germany] (Figure 1A). The implantable cuff and lead are an investigational device manufactured by Galvani Bioelectronics. The cuff is composed of two outer arms (with electrodes) and a middle arm (no electrode) (Figure 1B), designed to interface with the nerves surrounding the SpA. Two versions of the cuff were available in the study, the “6 mm” (which accommodated NVBs up to 7.8 mm in diameter) and the “7 mm” (NVBs up to 9.1 mm). The stimulation cuff diameter ranges were based on a previous study, which provided insight on the diameter of the human SpA using CT angiography (Brinkman et al., 2020). The proximal end of the cuff contains a connector for connection to the external pulse generator, the ISIS neurostimulator, *via* the extension cable. The ISIS Neurostimulator and accompanying software were used according to the manufacturer’s instructions.

### Procedures

All participants in this study were undergoing Minimal Invasive Esophagectomy with intrathoracic anastomosis (MIE) and creation of gastric conduit as previously described (Berkelmans et al., 2020; Janssen et al., 2021). During the abdominal phase of this procedure, a lymphadenectomy is routinely performed and the SpA is partially exposed. This procedure is highly similar to the laparoscopic procedure that was performed in pigs (Sokal et al., 2021). The stomach is mobilized before the splenic arterial loop is visualized to place the cuff. Due to the anatomical differences between human and pigs the spleen does not have to be retracted. At this point in the procedure, the PI and attending anesthetist determined if it was appropriate to conduct the experimental protocol. If the investigational procedure progressed, the splenic NVB was dissected from the attached soft tissue if not already done as part of the standard procedure. Following mobilization, the cuff was introduced through a trocar into the abdomen and was manipulated in the abdomen using atraumatic graspers and positioned around the splenic NVB. The other end of the cuff was connected to the external pulse generator *via* the extension cable. A single impedance measurement was taken to ensure proper function of the cuff. If there was a high impedance (>2.5 kΩ), the cuff was repositioned or replaced with a second cuff. Next the ultrasound transducer (L51K, Hitachi Medical Systems B.V., Reeuwijk, Netherlands), was introduced into the abdominal cavity through one of the trocars, to collect velocity profiles during the protocol. The intraoperative stimulation was performed according to the predefined stimulation scheme, with increasing amplitudes over the course of the trial. Total experimental procedure time (including additional dissection, cuff placement, optimization of Doppler imaging, stimulation, and cuff removal) was not allowed to exceed 40 min. The surgeon performing the implantations (ML) had completed a training program consisting of education on the use of the neuromodulation device and performing implantations in human cadavers.

**FIGURE 2:**
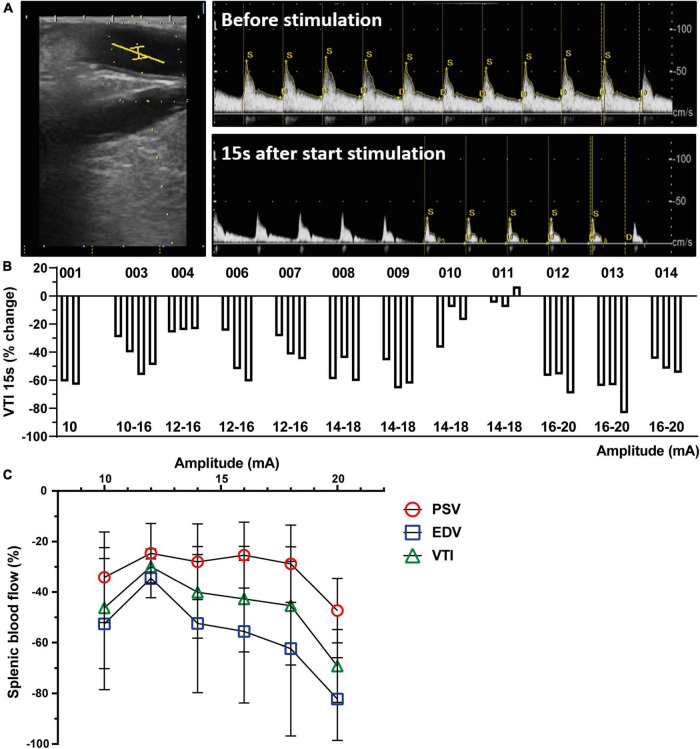
Splenic nerve bundle stimulation caused a reproducible splenic arterial blood flow change. **(A)** Representative ultrasound image of the SpA (left). The ultrasound probe was placed parallel to the splenic artery. A center point (yellow lines) in the artery was chosen to measure Doppler flow velocity profiles before (upper) and after start of stimulation (lower). **(B)** Change in blood flow (VTI) presented as percentage change compared to baseline 15 s after start of SpNS as presented per patient for each stimulation. The amplitudes of the stimulation are listed below. **(C)** Effect of increasing stimulation amplitudes on decrease in PSV, EDV, and VTI, averaged over all patients, compared to baseline 15 s after start of SpNS. SpA, splenic artery; SpNS, splenic nerve bundle stimulation; VTI, Velocity Time Integral; PSV, Peak Systolic Velocity; EDV, End Diastolic Velocity.

All stimulations used biphasic symmetric pulses delivered at 10 Hz frequency, 400 μs pulse width per phase, and 1 min duration. The current amplitude varied from 8 mA (3.2 μC) to 20 mA (8 μC), based on the results of previous studies and to investigate the effect of step-up stimulation on safety and target engagement (Gupta et al., 2020; Donega et al., 2021). Based on preclinical data, three stimulations were considered to be optimal (Guyot et al., 2019; Donega et al., 2021). For the run-in patients, 1–5 stimulations could be applied. The amplitudes were slowly ramped-up over the course of the trial with maximal 3 stimulations per patient in the main cohort. In Figure 2B and Supplementary Table 1 the different amplitudes used per patient are shown. Based on the physiological changes upon stimulation, the PI could decide to increase, decrease or maintain the stimulation amplitude, or not proceed with the next stimulation. Impedance measurements were taken before each stimulation to confirm good contact and functionality of the cuff. After each stimulation, splenic arterial blood flow and cardiovascular metrics had to return to baseline before initiating a new stimulation. After the last stimulation, a final impedance measurement was taken before the cuff was removed from the SpA NVB and abdominal cavity.

Splenic artery blood velocity measurements [Peak Systolic Velocity (PSV), End Diastolic Velocity (EDV), and Velocity Time Integral (VTI)] and were obtained using pulse wave Doppler. A laparoscopic ultrasound transducer (L51K, Hitachi Medical Systems B.V., Reeuwijk, Netherlands) was placed distally from the cuff on the splenic NVB. The probe was attached to an Ultrasound System from Hitachi (V70) and Doppler images were obtained according to the manufacturer’s instructions. The pulse wave Doppler signal was optimized intraoperatively by a trained investigator (DB). Briefly, the probe was placed in parallel to the SpA by the surgeon. A center point in the artery was chosen to measure arterial blood flow and angle correction (max. angle of 60°) and beam steering were used for correction of the Doppler signal. If a constant arterial flow curve was observed, PSV, EDV, and VTI were recorded as still frame flow velocity profiles and values from a minimum of three adjacent beat flow velocities were averaged. These values were recorded twice prior to each stimulation as a baseline, and twice during each stimulation (at 15 and 40 s). If the ultrasound probe moved between baseline measurements and stimulation, new baseline measurements were performed. All images and measurements were subjected to a quality review at the end of the study, with independent reading by a single investigator (RC). This included a review of image quality, selection of images for analysis, and evaluation of the measurements that were performed.

Heart rate and mean arterial pressure (MAP) were continuously monitored using a patient monitoring system (GE Healthcare, Chicago, IL, United States) as is standard care for MIE. In parallel with the blood velocity measurements, two baseline recordings for HR and MAP were manually documented from the system as well as during each stimulation at 15 and 40 s.

C-reactive protein (CRP) and leukocyte count were measured on postoperative day (POD)2, 3, and 4 as part of the standard care. CRP levels were determined in the morning (at 08.00 a.m.) by immunoturbidimetric assay (Roche/Hitachi cobas C system, Roche).

End of follow-up was defined as POD7, day of hospital discharge, or conclusion of any ongoing serious adverse events (SAEs)/AEs.

### Outcomes

The primary objectives of this study were to evaluate the safety and feasibility of the temporary placement, stimulation, and removal of an investigational cuff around the SpA NVB. Feasibility was demonstrated by the proportion of participants in whom the cuff was successfully applied to the splenic NVB and removed. Safety analysis evaluated all AE and SAEs that resulted from stimulation after cuff application, stimulation, and removal. SAEs were collected from informed consent until POD7 and AEs and device deficiencies from the day of the intraoperative procedure (day 0) until POD7. All AEs deemed unresolved or ongoing at the time of the POD7 or at the day of discharge assessment were monitored until the event was resolved, stabilized, or otherwise explained. AE were to be graded as mild, moderate, or severe. A serious event caused hospitalization or prolongation of participant hospitalization, a life-threatening illness or injury, a medical or surgical intervention to prevent life threatening illness, injury or permanent impairment to a body structure or a body function or chronic disease. Furthermore, the principal investigator and the sponsor had to determine whether the event was related to the study device, study procedure or surgical procedure.

A Secondary outcome was SpA BF changes as measured by the PSV, EDV, and the VTI. Exploratory outcomes included changes in HR and mean arterial blood pressure (MAP), white blood cell count at day 2–4 and CRP plasma levels at POD2–4.

### Statistical analysis

Due to the exploratory nature of this study, no power calculation for sample size was performed. After the run-in period (three patients), the number of ten participants was deemed sufficient to determine whether further research and development in this therapeutic area are warranted. To evaluate white blood cell count and CRP levels, patients in the trial were compared to patients that underwent MIE in 2019–2020 at the Catharina Hospital (*n* = 65). Propensity score matching was used to select specific controls for the trial patients. Because of the small sample size of the trial patient group, only three covariates were chosen to include in the propensity score matching analysis. These covariates included the occurrence of complications which are known to influence the postoperative inflammatory after surgery. A logistic regression analysis was performed on postoperative occurrence of any Clavien-Dindo complication ≥3, pneumonia and anastomotic leakage. The resulting propensity variables were used to select controls for the trial patients. A caliper width of 0.2 was chosen as recommended to minimize error rate. SPSS version 26.0 (IBM, Armonk, NY, USA) was used to perform propensity score matching and this resulted in a matched control for each of the trial patients (Austin, 2011). SPSS was also used to perform group comparisons on CRP and leukocyte levels using parametric or non-parametric tests as appropriate. Graphs were made with Prism 8.3 (GraphPad Software, La Jolla, CA, USA).

### Role of the funding sources

The sponsor of this study was Galvani Bioelectronics. The report presents the results of a sponsored study. The funding sources had a role in study design, data collection, analysis, and interpretation, and writing of this report. The corresponding author had full access to all the study data and the final responsibility to submit for publication.

## Results

Between November 2019 and September 2020, 37 patients were pre-screened for eligibility for this study. Of these patients, three patients were not eligible (history of pancreatitis, use of anti-inflammatory agents, or creation of colon interposition instead of gastric conduit), 6 patients could not participate (COVID-19 pandemic or logistic reasons that hampered cuff availability) and 14 patients did not provide informed consent. Fourteen subjects were consented to participate in the study; in one patient, the study procedures could not take place due to COVID-19 pandemic restrictions. This resulted in 13 patients participating in this feasibility study. Patients included in the study had an age range from 51 to 77 years and had a BMI range 19.1–33.8. The patient demographics are depicted in Table 1.

**TABLE 1:** Patient demographics and surgical data.

Patient	Age (years)	Sex	Preoperative status (ASA grade)	Surgery duration (min)	Blood loss (mL)
001	51	M	2	274	150
002	60	F	2	278	150
003	54	M	2	232	150
004	77	M	3	241	150
006	69	F	2	232	100
007	63	M	3	239	100
008	62	F	2	253	100
009	69	M	2	263	100
010	57	M	2	256	150
011	65	F	2	245	100
012	66	M	2	280	100
013	64	M	3	233	150
014	68	M	2	364	470

Patients’ lumen diameter measured by CT angiography scan ranged from 4.0 to 7.2 mm, and the 6 mm cuff size was selected for all participants prior to the surgery. In 10 out of 13 patients the preoperative CT measurements correlated with the cuff fit (Table 2). In three patients a loose cuff fit was observed.

**TABLE 2:** Artery diameter and cuff fit.

Patient	Artery diameter (mm)	Proximal arm fit	Middle arm fit	Distal arm fit	Comments by surgeon
001	5.1	Loose fit	Good fit	Good fit	Cuff seemed slightly too large
002	4.1	Good fit	Good fit	Good fit	No comments
003	5.5	Loose fit	Loose fit	Loose fit	Cuff is too large
004	5.0	Good fit	Good fit	Good fit	Cuff appeared to be somewhat loose
006	5.3	Good fit	Good fit	Good fit	No comments
007	5.7	Good fit	Good fit	Good fit	No comments
		Good fit	Good fit	Good fit	No comments
008	5.2	Loose fit	Loose fit	Loose fit	Better fit of distal arm
009	7.2	Good fit	Good fit	Good fit	Excellent fit
010	5.5	Loose fit	Loose fit	Loose fit	Very loose fit
011	5.2	Good fit	Good fit	Good fit	No comments
012	5.4	Loose fit	Loose fit	Loose fit	Loose fit of all three arms
013	4.0	Good fit	Good fit	Good fit	Good fit with additional fat tissue
014	6.5	Good fit	Good fit	Good fit	No comments

Placement of the cuff on the SpA NVB, applying stimulation and subsequently removing the cuff was successful in all patients. All cuffs were positioned in a single attempt except for the first patient where two attempts were made, and cuff placement in all patients was completed within 3 min, with 9 out of 13 placed in 1 min or less. In one patient, the implanted cuff needed to be replaced because high impedance values (>2.5 kΩ) were measured on the cuff after placement on the NVB.

All impedance measurements after implantation were within acceptable levels. All stimulations applied were of 1 min duration with 100% current delivery. Amplitude levels ranged from 8 to 20 mA (3.2–8 μC at 400 μs pulse width) with three stimulations applied to each individual participant, except for the first run-in participant who received two stimulations and the third run-in participant who received four stimulations. The starting amplitude in the first patient (001) was 10 mA. Because this caused a considerable decrease in blood flow (Figure 2B), a lower starting amplitude (8 mA) was chosen for patient 002. Thereafter, it was deemed safe to increase the starting amplitude in every three patients. Stimulation levels by participant are listed in Figure 2B with the accompanying blood flow changes (VTI) per stimulation at 15 s after start of stimulation and in Supplementary Table 1. During the study a total of 39 stimulations were delivered to the thirteen participants.

Eleven patients experienced a total of 24 AE, of these six were determined SAEs in six patients (Table 3). None of these were attributed to placement or removal of the cuff nor were they attributed to the stimulation, but they were all related to the MIE procedure.

**TABLE 3:** Adverse events.

Patient	Day	Event	Serious	Severity	Relationship to NVB stimulation
001	2	Mucus plugging	Yes	Severe	No
002	1	Tachycardia	No	Mild	No
	2	Dyspnea	No	Mild	No
	5	Pneumonia	No	Severe	No
	6	Anastomotic leakage	Yes	Severe	No
	7	Hypokalemia	No	Moderate	No
003	1	Reduced control over left arm	No	Mild	No
004	0	Hypotension	No	Mild	No
	2	Colon herniation	Yes	Moderate	No
006	6	Thrush	No	Mild	No
007	4	Atrial fibrillation	No	Severe	No
	5	Pneumonia	Yes	Severe	No
	18	Anxiety	No	Mild	No
	18	Excessive postoperative pain	No	Moderate	No
008	6	Pneumonia	Yes	Mild	No
009	5	Wound infection	No	Mild	No
	0	Excessive postoperative pain	No	Moderate	No
010	11	Pleural effusion	Yes	Moderate	No
	11	Hypokalemia	No	Moderate	No
	24	Delirium	No	Mild	No
011	1	Increased liver enzymes	No	Mild	No
012	–	–	–	–	–
013	–	–	–	–	–
014	0	Hyperglycemia	No	Mild	No
	1	Tachycardia	No	Mild	No
	3	Subcutaneous emphysema	No	Mild	No

Placement of the ultrasound transducer could be performed in all patients. Duplex ultrasound data were monitored from 36 stimulations in twelve participants. In one patient (002) the data was not recorded due to a technical issue with the ultrasound system. A consistent and reproducible decrease in splenic arterial blood flow was observed as represented by VTI except in one patient (011, Figures 2A, B). The blood flow decrease peaked at 15 s after start of stimulation and at 40 s (Supplementary Figure 1), the blood flow change decreased and optically returned to baseline levels typically within 60–180 s after cessation of stimulation. The reduction appeared to be stimulation amplitude dependent, and a correlation was observed between the stimulation amplitude and all blood flow metrics (VTI, PSV, and EDV at 15 s after start stimulation, Figure 2C), although statistical correlation is only found for the participants in the main cohort (excluding run-in patients, *R*-square value – 0.8247, *p* < 0.05). Cardiovascular endpoints were continuously monitored during the study procedure. Small changes in HR and MAP were observed, however, no clinically significant observations were made (Supplementary Figure 2). All observed changes returned to baseline within 1 min after the end of stimulation. Impedance measurements taken after stimulation and prior to cuff removal confirmed that the cuff was still electrically functional in all participants. Day of discharge and post-operative complications were captured but based on the small study population no conclusion could be drawn from this data.

**FIGURE 3:**
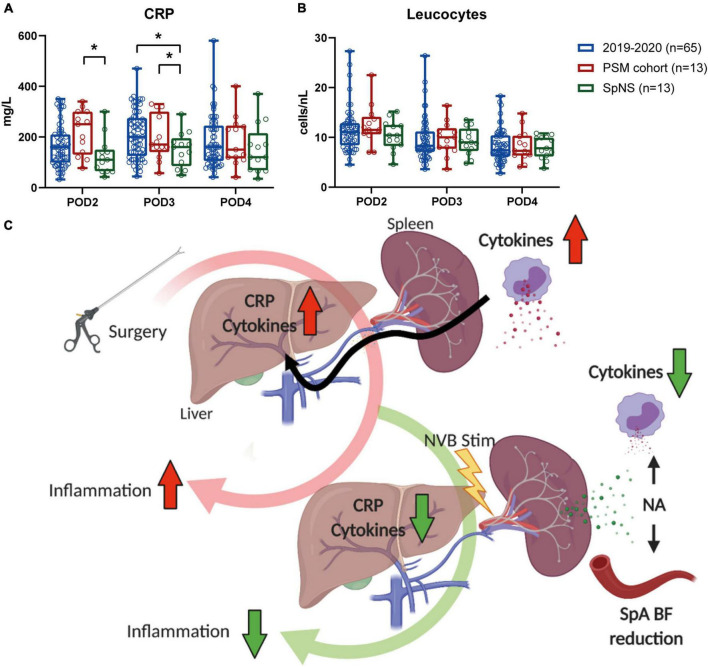
Postoperative inflammation was lower in patients that underwent SpNS compared to matched controls. **(A)** Plasma CRP levels and **(B)** blood leukocytes on POD2–4 in all patients that underwent MIE in 2019, patients that underwent SpNS and patients that were matched to SpNS patients based on the occurrence of major complications, pneumonia, and anastomotic leakage. Bars indicate mean and standard deviation. **(C)** Schematic representation of the putative mechanism of action. Surgical procedures trigger immunological responses. The accumulation of cytokines and the consequent production of CRP by the liver are biomarkers of postoperative inflammation and correlate with postoperative recovery. When stimulation of the splenic nerve is applied *via* a stimulation lead placed around the SpA NVB, norepinephrine (NA) is released within the spleen. The neurotransmitter causes contraction of the vascular bed with consequent transient decrease in splenic arterial blood flow that can be used as a biomarker of nerve engagement. At the same time NA binds to spleen resident immune cells and suppresses their ability to produce cytokines. *p < 0.05.

In a *post hoc* analysis, CRP plasma levels in the intervention group were significantly lower on POD3 when compared to a cohort of patients that underwent MIE (*n* = 65) in 2019 and 2020 in the same institution (150.5 ± 19.0 vs. 206.7 ± 11.6 mg/L, respectively; *p* = 0.043, Figure 3A). After propensity score matching (PSM), CRP plasma levels in the intervention group (*n* = 13) were significantly reduced compared to the PSM cohort (*n* = 13) on POD2 (124.2 ± 20.3 vs. 196.7 ± 24.7 mg/ml; *p* = 0.032) and POD3 (150.5 ± 19.0 vs. 220.5 ± 24.4 mg/L; *p* = 0.033). On POD4, no significant differences in CRP levels between the cohorts were observed (SpNS vs. PSM *p* = 0.413; SpNS vs. 2019–2020 *p* = 0.323). The number of leukocytes on any POD (Figure 3B) did not differ between groups. Baseline characteristics (Table 4) were not statistically different between matched groups.

**TABLE 4:** Baseline characteristics and postoperative complications of all patients that underwent esophagectomy in 2019–2020 (excluding trial participants), the participants in the pilot trial and a complication matched cohort from the 2019 to 2020 group.

	2019–2020 (*n* = 65)	Matched cohort (*n* = 13)	SpA NVB stimulation (*n* = 13)
Age, years (SD)	63.9 (9.6)	65.2 (13.0)	63.0 (7.0)
Sex, male (%)	54 (83)	9 (70)	9 (70)
BMI, kg/m^2^ (SD)	27.2 (4.7)	25.6 (4.1)	26.0 (4.8)
Preoperative status, ASA ≥3 (%)	17 (27)	2 (15)	3 (23)
Comorbidity (%)	44 (68)	7 (54)	6 (54)
Cardiovascular comorbidity (%)	33 (51)	7 (54)	3 (23)
Pulmonary comorbidity (%)	9 (14)	1 (8)	3 (23)
Diabetes (%)	9 (14)	0 (0)	2 (15)
Pathological tumor stage, ≥3 (%)	28 (44)	4 (31)	7 (54)
Surgery duration, min (SD)	248 (36)	243 (34)	262 (34)
Blood loss, ml [IQR]	100 [50–150]	150 [50–250]	150 [100–200]
Any 30 day complication (%)	35 (54)	7 (54)	6 (46)
≥CD3 (%)	17 (26)	4 (30)	4 (30)
Cardiopulmonary complication (%)	21 (32)	6 (46)	6 (46)
Pneumonia (%)	12 (18)	3 (23)	3 (23)
Anastomotic leakage (%)	6 (9)	1 (8)	1 (8)
Infection (including wound infection, %)	1 (2)	1 (8)	1 (8)

Values are shown as continuous (SD or IQR) or number (%). Comparisons were made using the *t*-test, Mann–Whitney U test, or Chi-square test as appropriate. BMI, body mass index; ASA, American Society of Anesthesiologists Classification; CD, Clavien-Dindo.

## Discussion

This first-in-human study demonstrated for the first time that applying a cuff around the splenic NVB and subsequent stimulation are safe, feasible and may alter the postoperative inflammatory response following MIE.

None of the AE were attributed to placement or removal of the cuff nor were they attributed to the stimulation, but they were all considered to be part of the normal postoperative outcomes for patients that undergo MIE. Although no AE (e.g., additional bleeding) related to the dissection of the SpA NVB were observed, it must be considered that the MIE procedure includes exposure or partial exposure of the SpA NVB as part of the obligatory lymph node dissection. In this pilot trial, the potential safety risks were mitigated by including a small number of patients, using one single experienced surgeon and providing extensive training on the device and surgical placement.

The cuffs used in this study were specifically designed to be delivered laparoscopically to the abdomen and fit around the human SpA NVB. In general, intraoperative cuff fit around the SpA was satisfactory, although a loose fit was observed in some patients, this is to be expected as the cuff was designed for chronic implantation where the NVB would be less dissected than in the MIE and NVB diameter could be affected by vasoconstriction or other factors. Nonetheless, SpNS led to an overall consistent and reproducible decrease in splenic arterial blood flow, which correlated with the stimulation amplitude in the patients participating in the main cohort and indicated nerve target engagement. Based on these observations and previous studies in pigs showing that a blood flow change in the SpA during SpNS directly correlated with the evoked compound action potential, it can be concluded that SpNS was successful in target nerve engagement in the current study (Donega et al., 2021). Reduction in SpA BF could therefore be used as a real-time biomarker for nerve engagement during intra-operative stimulation (Ayers et al., 1972).

Stimulation of the SpA NVB showed a minimal, variable effect on HR and MABP during MIE in patients under anesthesia. The changes were not statistically correlated to the stimulation amplitude. This is in contrast with the effects seen in terminally anesthetized pigs (Koopman et al., 2016). The differences can be because of the anesthetic agents used for a major surgery like MIE, or due to the anatomical and physiological differences between species.

Increasing experimental evidence has indicated that neural stimulation can reduce inflammation in acute and chronic animal models (Vida et al., 2011; Kressel et al., 2020). Although the current study was not designed and powered to show an effect on systemic inflammation, it was demonstrated that CRP plasma levels in the intervention group were reduced on POD2 and 3 when compared to a *post hoc* analysis in a PSM control group. A few studies have provided insight in the interpretation of CRP levels after major abdominal surgery (Platt et al., 2012; Adamina et al., 2014; Straatman et al., 2015). Postoperative CRP levels have shown a strong correlation with complications in patients undergoing open vs. laparoscopic colorectal surgery regardless of surgical approach (Straatman et al., 2018). Elevated CRP levels on the third POD after major abdominal surgery have been found to identify patients at an increased risk for developing major complications (Ayers et al., 1972). In this publication, a prediction model with a two-cut-off system is suggested. This system consists of a safe discharge criterion with CRP levels below 75 mg/L at POD3, and at 215 mg/L (probability 20%) to serve as a predictor of severe complications. Other studies mention a single cut-off value of CRP levels between 140–190 mg/L for an increased risk of severe complications (Adamina et al., 2014; Straatman et al., 2015, 2018). Although this data was based on several major abdominal surgeries, and not specifically MIE, CRP levels in the GAL1018 patients were 150.5 mg/L on POD3, indicating that neuromodulation could induce a clinical relevant reduction in CRP levels and reduce severe postoperative complications following major abdominal surgery.

Because feasibility and safety were the primary aim of this study, no power analysis was performed on the reduction of postoperative CRP levels. The true value of these exploratory results needs to be further confirmed in a statistically powered randomized controlled trial. Interestingly, the number of leukocytes, the cells mainly responsible for production of inflammatory mediators, did not show a difference between groups, suggesting that stimulation had a direct effect on the inflammatory process.

These findings are in line with what is expected based on the experimentally found underlying mechanism which has been previously shown (Koopman et al., 2016; Sokal et al., 2021) and which have been summarized in Figure 3C. Recently, it has been shown in chronically implanted pigs that SpA NVB stimulation using the same cuff as used in this clinical study, is well tolerated and reduces inflammation and promotes resolution of inflammation (Sokal et al., 2021).

## Conclusion

In conclusion, this first-in-human study represents a significant advancement toward the feasibility and safety of splenic neuromodulation in humans and supports the progress toward the development of bioelectronic medicine for patients with inflammatory conditions targeting the near-organ autonomic nervous system of the spleen. Future randomized larger studies are needed to substantiate the current results. Therefore, an acute randomized control trial in patients undergoing MIE is in preparation.

## Data availability statement

The datasets presented in this article, including individual participant data and a data dictionary defining each field in the set, are not readily available, as per ethics committee agreements. Requests to access the datasets should be directed to ML, misha.luyer@catharinaziekenhuis.nl.

## Ethics statement

The studies involving human participants were reviewed and approved by the MEC-U (Medical Ethics Committees United) Nieuwegein. The patients/participants provided their written informed consent to participate in this study.

## Author contributions

DB obtained the regulatory and ethics approval, recruited the patients, collected, analyzed, and interpreted the data, and wrote the manuscript. IG contributed to stimulation parameter development, collected, analyzed, and interpreted the data, and wrote the manuscript. PM supervised the cuff design and verification and validation (V&V) testing, and supported the external pulse generator programming for the trial and design of pre-clinical studies. SO developed the cuff and supervised the V&V testing. WJ supported the data analysis and interpreted the data. RC designed and performed the preclinical safety experiments in pigs and supported collection of the ultrasound data and interpretation of the results. TS developed the external pulse generator programming for the trial and inspected returned devices. DC was responsible for stimulation parameter development and involved in data analysis. EI was involved in development of implant procedure and performed safety analyses. RY supervised engineering of the Galvani System. GN recruited the patients, performed the surgeries, interpreted the data, and revised the manuscript. MV designed the study, obtained the regulatory and ethics approval, collected, analyzed, and interpreted the data, and wrote the manuscript. ML was involved in the design of the study, recruited the patients, performed the surgeries, collected, analyzed, and interpreted the data, and wrote the manuscript. All authors reviewed and approved the final version of the manuscript.
