# Supplementary material for: Splenic arterial neurovascular bundle stimulation in esophagectomy: A feasibility and safety prospective cohort study
Source: Front Neurosci. 2022 Dec 22;16:1088628. doi: 10.3389/fnins.2022.1088628 (PMC9817142; doi:10.3389/fnins.2022.1088628)
Supplement: Supplementary file 1 [file Data_Sheet_1.docx]

**figure S1.**

**
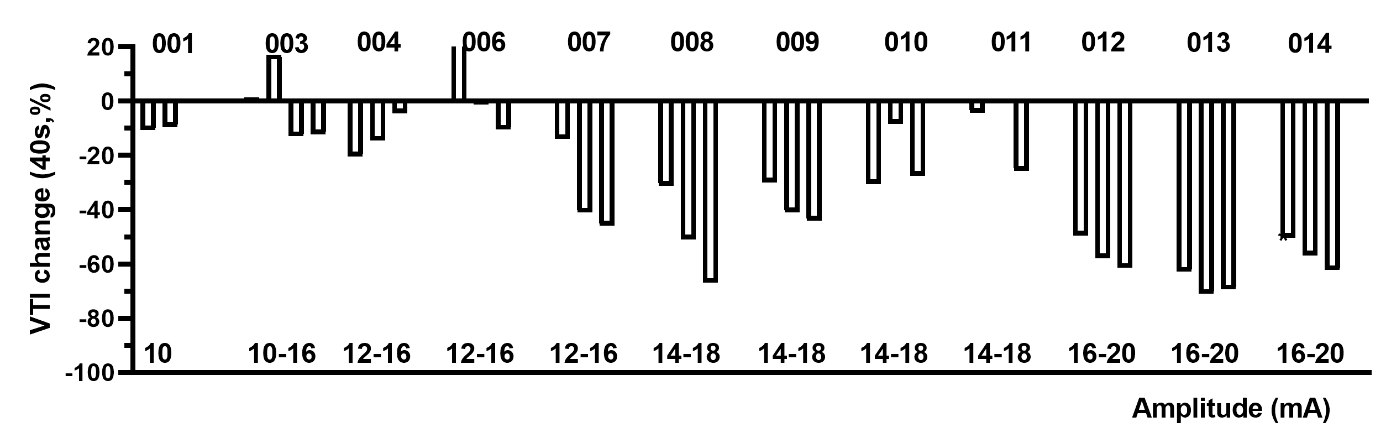
**

Change in blood flow (VTI) presented as percentage change compared to baseline 40 seconds after start of SpNS as presented per patient for each stimulation. The amplitudes of the stimulation are listed below.

**figure S2.**

**
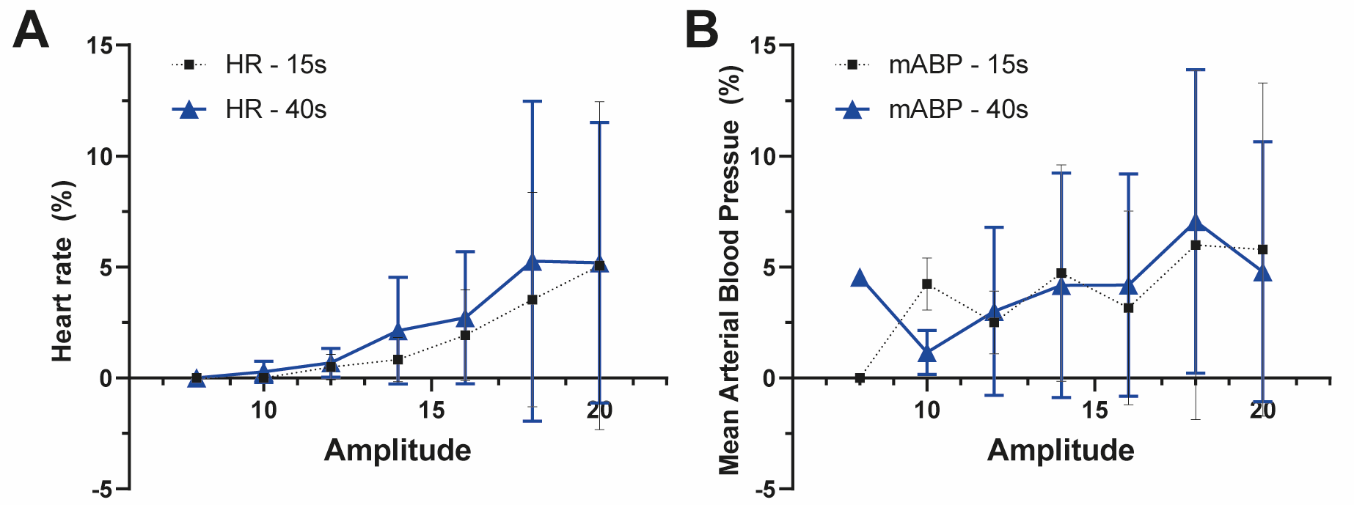
**

SpNS did not affect systemic hemodynamics. **A.** Change in heart rate (HR) and **B.** Change in mean arterial blood pressure (mABP) compared to baseline 15 and 40 seconds after start of SpNS.

**Table S1. Stimulation levels (amplitude)**

| **Patient** | **Stimulation 1** | **Stimulation 2** | **Stimulation 3** | **Stimulation 4** |
| --- | --- | --- | --- | --- |
| 001 | 10 | 10 | - | - |
| 002 | 8 | 10 | 12 | - |
| 003​ | 10 | 12 | 14 | 16 |
| 004 | 12 | 14 | 16 | - |
| 006 | 12 | 14 | 16 | - |
| 007 | 12 | 14 | 16 | - |
| 008 | 14 | 16 | 18 | - |
| 009 | 14 | 16 | 18 | - |
| 010 | 14 | 16 | 18 | - |
| 011 | 14 | 16 | 18 | - |
| 012 | 16 | 18 | 20 | - |
| 013 | 16 | 18 | 20 | - |
| 014 | 16 | 18 | 20 | - |
